# Supporting delivery of remote pulmonary rehabilitation across different healthcare contexts: A multi-national study

**DOI:** 10.1177/14799731241290518

**Published:** 2024-10-07

**Authors:** Narelle S Cox, Sarah Rawlings, Natasha A Lannin, Sarah Candy, Surya P Bhatt, Abraham Samuel Babu, Anne E Holland

**Affiliations:** 1 Respiratory Research@Alfred, 2541 Monash University, Melbourne, VIC, Australia; 2 568983 Institute for Breathing and Sleep, Melbourne, VIC, Australia; 3 Brain Recovery and Rehabilitation, Department of Neuroscience, 2541 Monash University, Melbourne, VIC, Australia; 4 Occupational Therapy Department, 5392 Alfred Health, Melbourne, VIC, Australia; 5 Te Whatu Ora, 637385 Health New Zealand, Auckland, New Zealand; 6 University of Alabama at Birmingham, Birmingham, AL, USA; 7 Department of Physiotherapy, Manipal College of Health Professions, 76793 Manipal Academy of Higher Education, Manipal, India; 8 Physiotherapy Department, 5392 Alfred Health, Melbourne, VIC, Australia

**Keywords:** Telerehabilitation, pulmonary rehabilitation, chronic respiratory disease, implementation, telehealth, rehabilitation

## Abstract

**Purpose:** This study aimed to understand factors that health professionals, from a variety of healthcare contexts and countries, believed support remote delivery of pulmonary rehabilitation (PR); and to develop a targeted intervention to support implementation of remote PR. **Methods:** A 3-phase participatory action-research process was employed, across three study hubs in three countries (NZ, India, USA), representing diverse healthcare delivery contexts. Phase 1 employed focus groups of health professionals working in PR; data were analysed qualitatively with transcripts coded against two implementation frameworks (Theoretical Domains Framework (TDF) and Consolidated Framework for Implementation Research (CFIR)). Findings informed development of an online toolbox to support delivery of remote PR (Phase 2), which was evaluated using semi-structured interviews (Phase 3). **Results:** 20 health professionals participated across all study phases. Factors considered to influence implementation of remote PR were consistent across diverse healthcare contexts and related to staffing availability, skills and confidence, and equipment and technology accessibility. An online toolbox provided support for enhancing knowledge and confidence, but was not able to address all implementation barriers. **Discussion:** Key factors to support clinicians deliver remote PR are common across different healthcare contexts, suggesting broader telerehabilitation implementation strategies may be applicable across healthcare environments.

## Introduction

Pulmonary rehabilitation (PR) is the primary non-pharmacological treatment recommendation for people with chronic respiratory disease.^1,2^ A program of exercise and education, underpinned by robust evidence, PR is typically undertaken in a group setting at a healthcare centre over 8-12 weeks. It effectively improves symptoms and function and reduces healthcare utilisation.^3^ However, despite robust evidence to support its use and the high burden of chronic respiratory diseases, a lack of available programs^4^ together with significant patient-related barriers to attendance^5^ means PR is grossly underutilised globally.^2^

Models of care that deliver PR remotely (i.e. telerehabilitation, rehabilitation delivery at a distance using information and communication technology (ICT)^6^) have been demonstrated to achieve equivalent clinical outcomes to traditional centre-based models^6^ and may reduce barriers to access. These remote models are associated with similar costs to traditional centre-based programs^7^ with greater program completion rates (exceeding 90%).^6^ Yet, despite the potential for improved PR access with remote delivery, and the clinical, health system and economic benefits ascribed to greater PR access,^8^ remote models of rehabilitation are not widely deployed. Clinical guideline recommendations relating to offering remote PR to patients vary in different jurisdictions,^2,9^ with a preference for centre-based PR as a first line treatment approach.^9^ Pre-COVID-19 remote programs were available in just 4% of Australian services^10^ and up to 30% of UK-based programs^11^; post-pandemic, widespread implementation of telerehabilitation for people with chronic respiratory disease remains limited.^12^ While patients report benefits associated with telerehabilitation including flexibility of program location, time and cost savings,^13^ healthcare professionals indicate a lack of knowledge and confidence to implement and adapt telerehabilitation programs for their local context as key barriers to effective program delivery.^14^

Previous research evaluating the real-word implementation of remote PR delivery has been limited to a select number of high-income countries and their health systems,^12,15^ with applicability in other settings, especially low-middle income countries, unclear. Such single centre evaluations typically do not account for setting context beyond the local organisation, or identify resources that will support and sustain scale-up processes, crucial factors for expanding intervention implementation to settings or populations beyond those initially studied.^16^ For telerehabilitation to be delivered on a broader scale, strategies to support program implementation that are applicable across healthcare jurisdictions may help overcome some barriers to the delivery of remote rehabilitation.

Therefore, the aims of the present work were to understand the factors that health professionals, from a variety of healthcare contexts and geographic regions, believed would support remote delivery of PR; and to develop a targeted intervention to support health professionals in the implementation of remote PR across a variety of healthcare settings.

## Methods

A participatory action-research process was employed across 3-phases. This allowed for gathering of practical information and perspective, taking action through intervention development, followed by evaluation.^17^ Three study hubs were used for targeting participant recruitment across three countries. These countries (New Zealand, India and USA) represent extremes of remote healthcare availability pre-pandemic ranging from ‘limited adoption, with infrastructure issues, and requiring the establishment of public policy’ [India], ‘telemedicine invention stage, but requiring technological sector support’ [New Zealand] to ‘front runner of telemedicine and technological innovation’ [USA]^18^; although the actual availability of telerehabilitation is not well documented.

The study hubs (Te Whatu Ora Counties Manukau, New Zealand; Manipal College of Health Professions, India; University of Alabama at Birmingham, USA) represented diverse healthcare delivery contexts, specifically: (i) a high income country with a significant burden of chronic respiratory disease amongst First nations people^19^ (New Zealand); (ii) a lower-middle income country where chronic respiratory disease is the second leading cause of disease burden and contributes 32% to global respiratory-related disability adjusted life years^20^ (India); and (iii) a high income country in a region with high prevalence of diagnosis, hospitalization and mortality due to chronic respiratory disease^21^ (USA). Healthcare professionals working in PR at the three study hubs, and their associated professional networks, were sent an email invitation to participate. Individuals who expressed interest in participating were provided with study information from the lead investigator (NSC) and sent an electronic link for completion of the online consent form. Approval for the study conduct and procedures was provided by the Monash University Human Research Ethics Committee (Project ID: 29483).

### Phase I: focus groups with health professionals

Hub-specific focus groups with up to 5 healthcare professionals per group were undertaken between September 2021–August 2022. The aim of the focus groups was to understand factors which clinicians, from a variety of healthcare contexts, felt would support implementation of remote PR. A discussion guide was employed (see Table S1), which was piloted with three PR clinicians (1 senior, 2 junior) from the local health service of the lead researcher in a mock online focus group. Focus group sessions were conducted remotely using videoconferencing and recorded for the purposes of transcribing audio content verbatim. Transcripts were coded to ensure that participants could not be identified from their responses. Focus groups were facilitated by one health researcher (NSC, female, PhD qualified) who is an experienced PR clinician with expertise in the delivery of remote rehabilitation, qualitative methodology, and focus group facilitation. There was no prior relationship between the researcher and participants. Participants were provided with a brief verbal biography of the facilitator prior to the commencement of the focus groups, and provided with an outline of the proposed running of the group and the aims of the session. A second researcher (SC, female, physiotherapist) was an observer for two of the focus groups providing additional perspective.

Focus group data were analysed independently by two researchers (NSC, SR) both experienced in the collection and evaluation of qualitative data. Participant quotes were coded against both the Theoretical Domains Framework (TDF)^22^ and the Consolidated Framework for Implementation Research (CFIR).^23^ The intent of using two coding frameworks was to identify individual-level (primarily TDF) and local organisational-level factors (primarily CFIR) that influence implementation of remote PR.^24^ Coding domains within both frameworks are not mutually exclusive, so a quote may be coded across both TDF and CFIR constructs as well as multiple domains within one framework. A random sample of 35% of the data was cross-coded by both researchers, with differences in framework assignment resolved by discussion. Double-coding was undertaken to ensure all focus groups and participants were represented in the data checking, and to establish intercoder reliability.^25^

### Phase 2: development of intervention to support delivery of remote PR

Using Phase I results, action points were generated for the development of an intervention. These action points were provided to participants in the form of an executive summary. The development of a free, online toolbox was proposed by the researchers, having considered the Phase I data, that would encompass the broad factors considered to support implementation of remote PR across all three healthcare contexts. Feedback was sought via an online poll (Qualtrics^XM^, Seattle, USA) with respect to executive summary content and proposed intervention development. Expert clinician-researchers then developed content for the online toolbox, and study participants were invited to use and review the online toolbox over a 1-month period.

### Phase 3: evaluation of intervention

Participants were invited to participate in a semi-structured, individual qualitative interview to provide their perspectives of the online toolbox intervention (https://prtelerehab.com) and reflect on the determinants of implementing a remote model of PR into their practice. Interviews were undertaken via video-conferencing. Audio recordings of interviews were transcribed verbatim. Transcripts were coded to ensure that participants could not be identified from their responses. Interviews were conducted by a healthcare professional (SR, female) with experience in delivering remote PR and qualitative research methodology, who was not involved in study Phase I nor in the development of the online toolbox. The interviewer was also not known to any of the participants prior to interview. An interview guide using opened-ended questions was designed to elicit information (Table S2).

De-identified interview transcripts were analysed by two researchers independently (SR and NSC). Through a process of reading and re-reading,^26^ line by line thematic analysis was performed to identify descriptive codes from the data.^27^ A data-driven code book was then developed using an initial sample of four interviews, and codes discussed between the two researchers for consistency of interpretation and reliability of application.^28^ The code book was employed for the analysis of remaining interviews, with coding being an iterative process whereby additional codes were added, removed or modified, as required, in keeping with a reflexive thematic analysis.^29^ As in Phase I, 35% of the data was cross-coded by both researchers to ensure consistency.^25^ On completion of data coding, related codes were collapsed to create major themes and subthemes. Themes, and their descriptors, were discussed between the researchers until consensus was reached. Quotations were extracted from the transcripts to provide supportive data for each theme.

## Results

### Phases I & II: focus groups and intervention development

Twenty participants (*n* = 14, 70% female) from NZ (*n* = 10, 50%), India (*n* = 6, 30%) and USA (*n* = 4, 20%) participated in 8 focus groups, each with between 1 and 5 participants and median (interquartile range [IQR]) duration 68 [56 to 73] min. The healthcare context in which participants primarily worked was centre-based (hospital outpatient *n* = 9 (45%); community facility *n* = 7 (35%)), with four participants exclusively delivering home-based PR programs (with or without ICT). The majority of participants (*n* = 17; 35%) worked in programs that saw between 50 and 200 patients each year, with a typical program lasting 6–8 weeks (*n* = 12, 60%) and offering 2 (*n* = 11, 55%) or 3 (*n* = 6, 30%) sessions/week. Only 10% (*n* = 2) of participants had no prior experience of delivering PR remotely (Table 1). Participants from New Zealand all worked in health services which operate within a publicly funded system. Participants from India worked across both tax-funded public hospital facilities and multi-payer private settings. Participants from the US all worked within a mixed system where healthcare services may be funded publicly (government Medicare and/or Medicaid) or privately (private health insurance).

**Table 1.** Participant characteristics.

Characteristic	*n* (%)
Profession	
Physiotherapist/physical therapist	15 (75%)
Respiratory therapist	1 (5%)
Exercise physiologist	3 (15%)
Nurse	1 (5%)
Physician	0 (0%)
Years of practice	
<1 year	0 (0%)
1 to <3 years	5 (25%)
3 to <5 years	3 (15%)
5 to <10 years	2 (10%)
>10 years	10 (50%)
Highest education	
Undergraduate	6 (30%)
Post-graduate diploma	1 (5%)
Masters	11 (55%)
PhD	1 (5%)
Not stated	1 (5%)
Telerehabilitation modality experience, *n*	
Telephone	16
Videoconferencing	15
Text message	7
Website	2
Mobile application	3
Mail/post	7
Email	3
Social media	3

**Figure 1.**
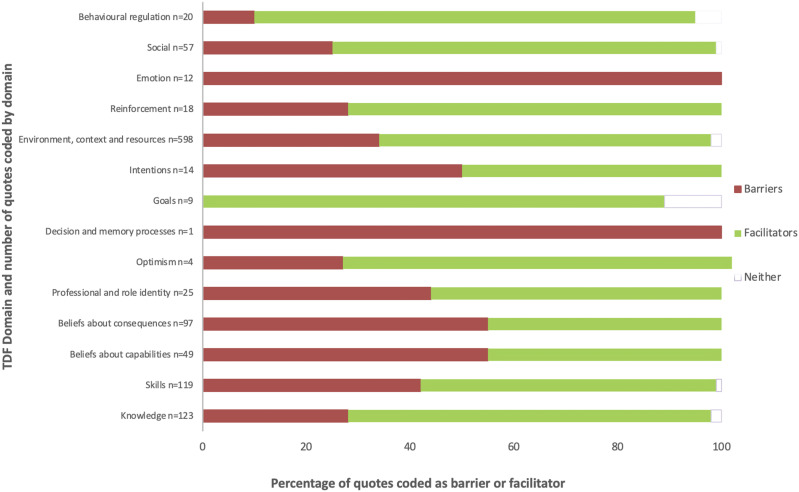
Participants quotes coded to TDF domain by barrier and facilitator.

A total of 616 individual participant quotes were extracted from the focus group transcripts and mapped to the TDF and CFIR as factors considered to influence remote PR delivery. All 12 domains of the TDF, and 5 domains of the CFIR, were represented including at least one quote attributed to each domain (Figure 1, Table 2). Despite participants working across diverse healthcare contexts, the factors considered to influence implementation of remote PR were consistent, and mutually inclusive across the TDF (see also Supplemental Material) and CFIR.

**Table 2.** Phase I representative quotes by TDF domain.

TDF domains	Representative quotes	Barrier/facilitator
Knowledge	‘We’re seeing a patient, and not only just the patient but the background and the living room or wherever they are, you can get a lot of like non-verbal sort of understanding of how they’re coping.’ NZ08	F
	‘A lot of people are really keen to have the exercises but they just don’t know how to use zoom, and it just stops them from exercising with us.’ NZ05	B
	‘So, if someone else wants to start with tele-pulmonary rehab, they just have to go through the guidelines and they know what has to be done and how to go ahead about it, and then they are there. So that’s what I really missed when I started with tele-pulmonary rehab. And I think so it would be really interesting to have, uh, like a good set of guidelines and to follow them accordingly.’ India 02	B/F
	‘But I think to understand enough and to be able to provide the service to, you know, suitably, you would need some experience, and you wouldn’t want to be a new grad.’ NZ06	F
	‘Clinically prepared staff members are very important, um, and I think experience is huge, but can it be done with less experience but a very good manager of those less-experienced people…’ US03	F
	‘Definitely people are more relaxed and the rapport building is better at home and in their environment rather than ours. Um, it gave me much more information on what she was able to do.’ NZ07	F
Skills	‘We deal with patients that their literacy, their ability to operate educational materials and things sent to them. It’s difficult right now.’ US 03	B
	‘We had other people that say they would love to do it but they don’t know how to use zoom or they don’t know how to use the phone.’ NZ07	B
	‘If someone shows me how to do it, I can do it [laughs], but to wrap my mind around a… how do you really get these, ah, platforms to work fully to my advantage, I gotta have someone young to say, oh, this is how they open it, this is how they do it, and this is how you would respond back to ‘em. I got the clinical background but I don’t understand the, ah, technological aspect of it.’ US03	B
	‘It is a lot more work than the centre-based group, because with every single person we enrol we have to do, like, a practice session, so we can make sure they’re confident where the mute is in the video call.’ NZ01	B
	‘The staff member that can deliver remote rehabilitation is someone that already has a grounding in managing these kinds of patients in a rehabilitation setting.’ India02	F
Professional role and identity	‘Because, you know, we’re clinicians, we shouldn’t really be having to spend so much time doing all this techy stuff.’ NZ01	B
	‘… a lot of times the calls are not related to rehab. The calls are related to, you know, I am feeling slightly down, and I feel slightly breathless. What do you think I should do? Should I come back now? And I think that’s an important link, because we are able to then speak to the treating physician, get some input from them, and then give them some advice, because it’s hard to get the physician on the phone, but it’s easy to get us on the phone.’ India 05	F
Beliefs about capabilities	‘I think I’m better, ah, speaking with a person when I’m face-to-face because you, you may miss a lot of things, um, just being via video the whole time.’ US 02	B
	‘As a clinician, it would be really hard, in a class of 16 or more, to, in real-time, be keeping track of all those people and kind of what they’re doing.’ NZ09	B
	‘Definitely were maybe underestimating what they possibly could do at home just from that safety side.’ NZ01	B
	‘Depending on their symptoms, or depending upon the conditions. So, probably, ah, three to five patients is something which I believe, because there I can do justice for each of them.’ India03	F
	‘If there is a choice then I would definitely go for the video conferencing. Um, I feel much more confident to prescribe exercise if I’ve just seen what someone looks like and what they can move like, and if they can do a sit to stand, and, um, and, …, get a bit of an idea about the surroundings.’ NZ07	F
	‘It does show you what you can achieve if you do have all, all the resources and, and people can, um, can do it. So I think you can achieve quite a lot.’ NZ08	F
Optimism	‘Maybe somewhere down the road, we can deliver a very cost-effective rehab through, ah, teleservices to get to more people. Because clearly, they’re not gonna come to the facility, especially if you’re in an urban area where you gotta go downtown and find parking, get out of the car, go into your facility. If you could do it in their house and they’re willing, I think they’ll be more likely to participate.’ US03	F
Beliefs about consequences	‘I do feel more comfortable pushing a patient when I’m standing right there, and I have a crash cart available. And I have a doctor on call, and I can get them help when they need it.’ US 02	B
	‘I think that there needs to be a combination. I think we need to offer tele rehab and offer centre-based, especially, you know, for those that are younger and work. Um, I think we need… if we’re going to have both programs which is what I suggest, we need more staffing. … It’s, it’s too difficult to be doing centre-based and then also, um, running tele rehab. Like, you definitely need more staff.’ NZ 03	B/F
	‘I like to see progress. And that often comes through outcome measures. And so, that’s the hard thing about doing a thing at home, is actually, how do you judge that you’re getting better? That’s the biggest frustration, I would say.’ NZ 02	B
	‘The likelihood is they won’t go home and do it. Maybe, maybe 10%, 20% will, but the 80%, unless you’re standing there and you’re looking at me and I’m looking at you [laughs] saying, all right, we’re gonna do this, they will say, you know, … they just don’t want to do it, they just won’t do it.’ US 03	B
	‘I think one of the big things that hangs over the top of us is the fact that we, we want to do no harm.’ NZ10	B
	‘It engages the patients to where they’re more involved with their treatment, and I think that definitely leads to better outcomes as well.’ US 04	F
	‘I think that there is perhaps an opportunity with tele-health to make things a little bit more, um, tailored.’ NZ 07	F
Reinforcement	‘It is a lot more work than the centre-based group, because with every single person we enrol we have to do, like, a practice session, so we can make sure they’re confident where the mute is in the video call.’ NZ01	B
	‘So that’s the biggest advantage that I’m able to reach more patients. Able to reach remote areas. Able to deliver good quality care. And compliance is better.’ India 02	F
	‘That’s been positive in that the people that have chosen the home-based arm are really happy. I’ve only managed to reassess one of the home-based ones, but she loved it, she thought it was amazing.’ NZ06	F
Intentions	‘We need to have a homebased service, but I’m not sure what the optimal way of delivering that is. And I think we fall back to our comfort zone of a centre based programme because it’s what we know.’ NZ07	B
	‘I think in an ideal world that first assessment would be face-to-face.’ NZ 07	F
	‘I think it’s about choice, I think a lot of the negativity for us comes from lack of choice.’ NZ06	F
Goals	‘And first we should need to ask the patient what their goals are. What do they want to achieve from this program?’ India 06	F
	‘So I would get a WhatsApp message saying, this is what I did last week. And I’d say, excellent, do 500 more. And, ah, you know, and that would work actually.’ India 05	F
Memory, attention and decision processes	‘I find that quite, um, a big judgement call, to make sure that people are going to be safe. Um, because at the moment, there’s no strict criteria for it.’ NZ10	B
Environmental context and resources	‘Everyone seems to have a smart phone pretty much, um, but they, um… digital literacy is very low. Um, and, so that means like even with our zoom exercise class, some days some people are fine and some days they can’t their camera to work, or they’re trying to do a zoom exercise on a tiny little camera and they can’t be too far away from it because then they can’t hear and see us’ NZ07	B
	‘So many of our patients, ah, um, you know, English isn’t their first language and it really rules… it doesn’t completely rule them out. There is, um, ability to get an interpreter. It’s just not as easy to get an interpreter. And so that opens up another big challenge for people that may want to attend zoom education sessions or the exercise classes but may not be English speaking.’ NZ08	B
	‘The biggest problem I’ve found was that, um, out of 10 people, only five had the technology to attend a zoom class.’ NZ10	B
	‘Not currently. Not with our current team as it stands, so probably like maxed out in terms of what we’re delivering, or, our perception is that we would be maxed out…if we wanted to add in other telerehab or home-based stuff, then where do we fit that into the jigsaw?’ NZ04	B
	‘We didn’t have a platform to see multiple patients, which is, kind of, scary to think about from the standpoint of how do you handle if you have four people and two people go bad while you’re on the… on the screen.’ US03	B
	‘Even if we had the perfect system in place where every single patient with every qualifying diagnosis went to pulmonary rehab, the way we deliver here, even in the clinics, in my clinic right now, we couldn’t accommodate 75% of the patients that are eligible.’ US03	B
	‘There has to be a different set of guidelines. Um, because you’re right from technical issues, how to manage emergencies, everything, even the patient ratio, patient and therapist ratio. Everything needs to be more precise in the tele-pulmonary rehab, as compared to in person.’ India02	B
	‘We had to be creative in that way as well, so we tried to do all video with our patients and a lot of times, if the connectivity was poor, we would move towards more of a telephone based. Ah, I’ve texted, emailed, done everything I possibly could to communicate with the patient before. Ah, but that was probably one of the biggest challenges, was connectivity.’ US02	B
	‘Like [other participant] said, if you were at home and your kids weren’t here, and you had the, you know, the printer set up, and the proper computer with zoom, then you know, it actually could be relatively enjoyable. Because you’d take away all those other unsatisfactory things with work, like travel and parking and, and all of that, so it could work well for staff.’ NZ04	F
	‘As the clinician being able to put your eyes on the patient is huge because reading a clinic note or anything like that. Even seeing them, um, in video, it’s just not the same as being able to see them. So, but either some sort of in-person component, I know you mentioned the clinician having an initial visit, um, with the, the patient. I think that would be beneficial.’ US02	F
	‘The compliance is way much better in tele-pulmonary rehab as what it was centre-based. Because the barriers faced then was, one, is travelling. That was the biggest issue. Uh, patients were dependent on oxygen, it was very difficult for them to travel, commute.’ India02	F
	‘I think if we had some sort of tech navigator...if we had access to someone who could, um, that we could refer to, that would be able to take the time to set up the person on their device.’ NZ04	F
	‘So, we, ah, had some, we had their address if something happened and we needed to, to, um, to access some emergency services, we would do that.’ US01	F
Social influences	‘A handful of people requested, um, doing the telephone because I don’t think they felt comfortable being on a video. I think they thought it was awkward because they’ve never done it before.’ US02	B
	‘Social connections for our current demographic are fundamentally meant to happen in person...Unless they have, you know, grandchildren, someone to sort of help them, um set them up. I guess, fundamentally, they probably still won’t be really comfortable with it. Because, at the end of the day, they’d rather… You want to make those connections in person.’ NZ03	B
	‘I think, they actually, the same way they are in a group setting in a centre-based were encouraging to each other. Because invariably one would finish before the other and they were able to see them and after three or four visits they got to know each other….they knew that this is the person I’m going to exercise with. So, there’s still that social component for them which was great.’ US02	F
Emotion	‘And I’m not that good, but I’m not, I’ve got more experience than some of these patients. So I really feel for them, because it makes me so mad when it doesn’t work.’ NZ06	B
	‘Yes I was nervous about it because I’d never done it before. And so, you rely heavily on your equipment and, ah, the things that you’ve done over and over and over again when you’re at your centre-based setting.’ US02	B
	‘I was quite uncomfortable, um, prescribing that (exercise based on a telephone assessment).’ NZ03	B
Behavioural regulation	‘I think it just engages the patients a little bit more in their care, even if it is remote, and I think there’s a, a remote component, because we can perhaps individualise that care for somebody who normally wouldn’t come into a centre.’ US04	F
	‘The compliance is way much better in tele-pulmonary rehab as what it was centre-based. Because the barriers faced then was, one, is travelling. That was the biggest issue. Uh, patients were dependent on oxygen, it was very difficult for them to travel, commute.’ India02	F
	‘I like that it could be a bit more sustainable, in that I think there’s quite a bit of a drop off with pulmonary rehab…So maybe you’re going to set up more of a behaviour change if you get them doing it home, at, um, in an environment that is sustainable and they can carry on with, and they’ve found that inner drive of self-motivation to do it.’ NZ04	F

*Individual* factors associated with delivery of remote PR included sufficient staffing with appropriately experienced personnel. In addition, clinical skills in the management of people with chronic respiratory disease, and PR, were viewed as critical to being able to deliver remote PR effectively and safely. The use of video technology for delivery was felt to improve comfort and confidence of health professionals in their ability to conduct remote exercise sessions, evaluate patients, and monitor safety. Making use of team members or other service providers with previous experience of remote rehabilitation delivery as a learning resource, and ensuring understanding of procedures for managing emergencies or technological issues were key factors to ensuring successful remote delivery. Health professionals identified the need for patients to have an appropriate home environment for exercise, and access to and ability to use relevant technology as crucial.

*Organisational* factors considered to influence remote PR delivery were adequate program funding or reimbursement, and availability of infrastructure and resources. To support remote PR delivery health professionals would ideally have access to IT support in the form of a ‘technology navigator’ to relieve them of the burden of teaching and troubleshooting technology issues and address the lack of confidence with technology expressed by many participants. Although audio-visual interaction with patients via video was the preferred means of remote PR delivery, it was acknowledged that resources for video interaction were not readily available to all patients or health services.

**Figure 2.**
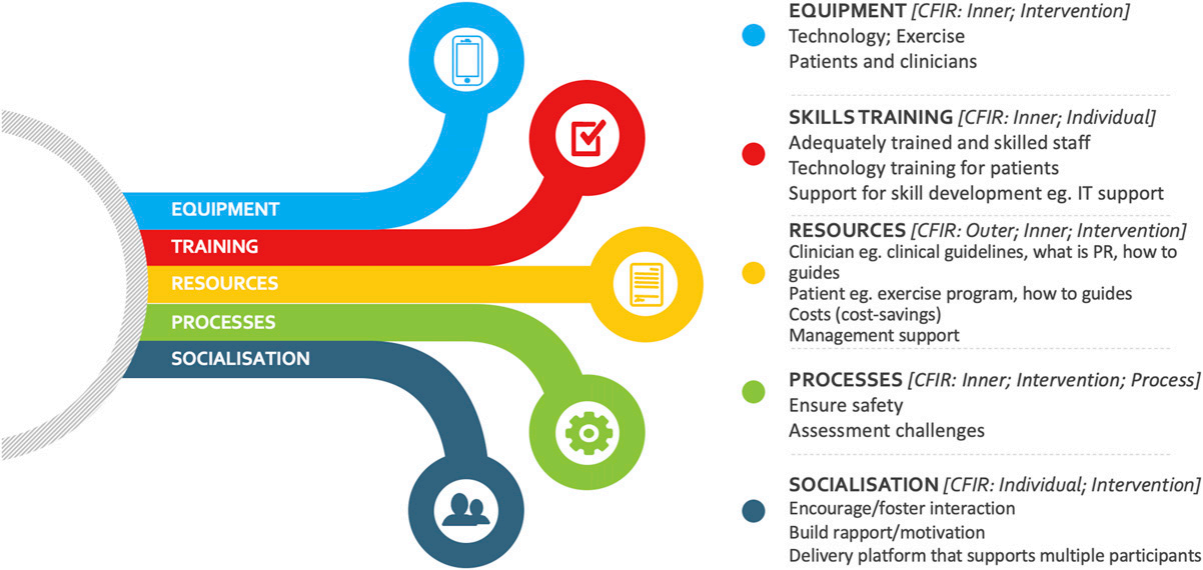
Executive summary of focus group data mapped to CFIR.

At the conclusion of the 8 focus groups, no new insights were apparent. An executive summary of these findings was prepared and disseminated to participants for review and feedback (Figure 2). Broad factors considered to support implementation of remote PR across all three healthcare contexts to be considered in toolbox development were categorised as relating to: (i) Equipment – type, availability and use (*CFIR domains: Inner, Intervention*); (ii) Skills training – for delivery of program components, and using and troubleshooting technology (*CFIR domains: Inner, Individual*); (iii) Resources – management, financial and practical to support program delivery (*CFIR domains: Outer, Inner, Intervention*); (iv) Processes – to foster confidence and competence in program delivery and ensure safety (*CFIR domains: Inner, Intervention, Process*); and (v) Socialisation – considering models that allow for a virtual group environment and visual interaction to create opportunities for peer-support and rapport building (*CFIR domains: Individual, Intervention*).

14 participants reviewed the executive summary and provided feedback via the online poll. The concept of an online toolbox to provide clinicians with resources and support for the delivery of remote PR was supported by 93% of respondents. Additional content suggestions included information on assessment strategies, patient monitoring, and locally and culturally adaptable resources.

### Phase III: intervention evaluation: individual interviews

The online toolbox (https://prtelerehab.com) was made available to all participants for review. 11 participants (55% total sample) agreed to an interview (New Zealand *n* = 4, USA *n* = 2, India *n* = 4; Physiotherapist/physical therapist *n* = 7, Exercise Physiologist *n* = 2, Nurse *n* = 1). Interview duration was median [IQR] 32 [18 to 46] min and data saturation was achieved.

Three overarching themes were identified from the interviews: (1) The toolbox is user friendly and valuable but challenges to remote rehabilitation delivery remain; (2) The toolbox can support knowledge gain but confidence to deliver remote PR and acceptance of these models is an issue; and (3) The future of remote PR is exciting, but further expansion of the toolbox may be warranted. Representative quotes identified to each theme are presented in Table 3.

**Table 3.** Individual semi-structured interviews, representative quotes by theme.

	Subtheme	Supporting quote
Theme 1: The website is user friendly and valuable but challenges to remote PR delivery remain	Value	‘A phenomenal resource. And I think, um, this is the way of the future.’ NZ06
		‘The moment you open a website, it’s like it is very positive and beautiful colours, ease of navigation. You have all the homepage resources, everything mentioned at one point.’ India02
		‘I thought the toolkit handles the fundamentals.’ US03
		‘It’s one of those things if you could have somebody go onsite and go over everything that you would need which is just virtually impossible. It’s not sustainable, but I think your website does the next best thing. It gives you that initial structure’ US04
	Easy to use	‘It’s definitely easy to, read and digest for somebody that doesn’t have experience in telerehab…to be able to, to set it up, it is quite clear in that regards.’ NZ08
		‘I thought it was nice and that, but even for me, I mean, and I’m, you know, again, showing my age here, but I’m not the most technologically savvy person but I, I found the navigation fairly easily, easy on the website.’ US04
		‘It will be accessible on the web, which means that then clinicians and patients and anyone can really access that information.’ India02
	Ongoing challenges	‘I don’t know that we had our ratio correct. We definitely could do with some more staff to provide more classes.’ NZ06
		‘Lots of our patients do not have the infrastructure’ India01
		‘There is the access to the technology, and then the ability to use the technology’ NZ06
		‘…Good technology. Um, technology that works. Um, equipment, a way for, to assess your patient at home.’ US01
		‘It’s just straight up reimbursement. I mean, how much are we going to get paid for doing this because, you know, you put it bluntly the hospital needs to see a check.’ US04
		‘Unfortunately, rehab is not revenue generating as much as, uh, other areas are, so there’s always going to be a little, uh, challenge with proving a business case for rehab, uh, from a return-on-investment perspective.’ India05
Theme 2. The toolbox can support knowledge gain but confidence to deliver and acceptance of remote PR is an issue	Website as a knowledge resource	‘What I liked is, uh, there are a lot of checklists which come handy even if I want implement it here. It provides you a template of what you may have to work on. It is quiet easy…as long as you’re giving it as a template I think it has to be modified to every particular patient, or even every set up.’ India01
		‘I really liked that the documents, there were some documents on there to use to help get started. I think some assessment tools and questionnaires, things like that. I really liked that.’ US01
		‘I think that the resources list that you’ve got is really comprehensive, so it’s quite nice, like [a] one-stop shop.’ NZ08
		‘There was some good resources in there around activity diaries and having all the different um exercise tests. I thought the information around emergency plan was really well done.’ NZ07
	Confidence/acceptance	‘If you have the knowledge base and you feel comfortable with the patient population experience you can probably, you can use the toolkit.’ US03
		‘Assessing the patient myself would give me confidence. ‘Cause I know I’ve asked all the questions.’ NZ06
		‘I think the healthcare people love that face to face still because that’s how we were trained.’ US03
		‘I think people have gone back to preferring the centre-based model.’ NZ08
		‘And would the clinician that has the experience to be able to operate the technology, deliver it quickly, would they find doing remote rehab in their wheelhouse?’ US03
		‘Readiness to change from the patient’s perspective is really poor.’ India04
Theme 3: The future of remote PR is exciting, but further expansion of the toolbox may be warranted	Future toolbox expansion	‘Maybe one if you want some referral mechanism, you can add.’ India04
		‘If it can be multilingual. Because not everyone might understand English.’ India02
		‘If we could get to see someone doing it, at least say couple of times or through some videos or on an online training programme or something…that would make us more confident.’ India02
		‘Maybe the, to reflect some low resource aspects, because even in Australia there are low resource settings.’ India05
	New directions and future intentions	‘We’ve also implemented that all the people that signing up to telehealth, they will get a call from our telehealth team.’ NZ05
		‘We are in fact launching this traineeship now, under our banner, under our company, because that’s something which we are lacking. We are not able to get as many physios now who are specialising in master’s cardiopulmonary’ India02
		‘In my ideal service, we would have a weekly telephone call, as well as the zoom exercise class, so that you got a little bit of individual coaching.’ NZ07
		‘We are trying to see if we can pre-record for certain patients’ India02
		‘Doing evening clinics…so those that cannot attend right during the day. It’s nice to be able to offer more options.’ NZ08
		‘We should be trying to do a home visit.’ NZ07
		‘The use of some remote monitoring is something that I’m just, kind of, trying to research a little bit more. Whether it’s use having a guideline, or some sort of, yeah, something out there about what’s going to be the best thing moving forward.’ NZ07

All participants reported the toolbox to be easy to use and perceived value for those with and without previous experience of delivering remote PR. The breadth of resources provided, including patient-facing documentation and service development templates as well as links to evidence-based literature, contributed to the perceived comprehensiveness of the toolbox as a training and service development resource. In future toolbox updates, participants recommended language translation capabilities and increased use of video resources as desired content. Despite the perceived usefulness of the toolbox in supporting clinicians to deliver remote PR, participants acknowledged ongoing barriers to remote service delivery including limited staffing resources and variable acceptance of remote rehabilitation by both patients and staff. Although challenges to implementation of remote delivered PR remain, clinicians described future goals for their services to further improve patient access to remotely delivered rehabilitation including offering evening programs and providing individualised coaching within the context of a rehabilitation program.

## Discussion

This study reports on factors which healthcare professionals working across different healthcare contexts around the world considered would support the remote delivery of PR. The findings informed the development of a free, online toolbox to support the implementation of remote PR (telerehabilitation). In a group of clinicians, almost all of whom had telerehabilitation experience, key factors that impact delivery of remote rehabilitation were not different across countries. Availability of appropriately skilled staff and sufficient workforce capacity, access to reliable technology and exercise equipment, and resources and support for skills development were all factors perceived to impact the implementation of remote rehabilitation. The online toolbox was well received, and provided clinicians support for enhancing knowledge and confidence, but challenges to remote rehabilitation delivery remain.

The use of two implementation frameworks in this study allowed for detailed exploration of behaviour change factors, within and by organisations and individuals,^30^ that could support the implementation of remote PR. That key facilitators of remote PR delivery were common across all healthcare contexts highlights the potential for strategies to support telerehabilitation implementation having application across multiple healthcare environments. To date, evaluation of implementation strategies for remote PR delivery have tended to focus on a single modality or health system model.^12,31^ The choice of an online toolbox, informed by the findings of study Phase I, as a means of providing implementation support creates an easily accessible resource, irrespective of healthcare jurisdiction, and was considered to act as a ‘one-stop shop’ for remote PR resources. Health professionals have previously described such online resources as being important for making information readily available, and supporting knowledge gain and training across the spectrum of workplace experience but particularly for more junior staff.^32^ As well, the concept of an online toolbox can help serve as a catalyst for service change.^33^ In a study of health professionals primarily delivering remote neurological rehabilitation, an online toolkit helped participants to demonstrate the utility and importance of remote rehabilitation to organisational management.^33^ In this way the toolbox may serve as a means to lobby, in particular, for organisational change by highlighting the resources and program components, with relevant adaptation to local context, identified to support remote PR delivery.

The use of videoconferencing to support remote PR delivery was favoured by participants. The ability to see patients via video was felt to create rapport and encourage clinician confidence by enabling a visual element for monitoring and evaluation. It was, however, acknowledged that videoconferencing was not available to, or preferable for, all patients. There is increasing evidence that patient preference for remote healthcare consultations varies by modality, with people of older age, lower socio-economic status, and female sex along with minority groups and those from non-English speaking backgrounds typically less likely to choose video-based interactions.^34^ Perhaps paradoxically, while video for remote rehabilitation was preferable, clinicians lacked confidence with operating and trouble-shooting technology equipment and saw this as a significant barrier to implementation of telerehabilitation. Lack of confidence with technology is a long-standing concern of clinicians for remote service encounters,^14,35,36^ and affirms that strategies to support remote PR delivery need to not only foster knowledge and skills development but require institutional support for cross-discipline collaboration specifically with ICT providers. While the online toolbox provides theoretical support for the use of videoconferencing to deliver telerehabilitation, its capabilities do not extend to hands-on skills practice. In future, blended learning opportunities, incorporating skills practice, simulation and ‘real-life’ observation, may better support health professionals confidence to integrate telehealth-delivered care into clinical services.^37^

The COVID-19 pandemic saw the rapid scale-up of remote healthcare delivery.^38^ Despite the pandemic inspired removal of many barriers to remotely delivered healthcare,^39^ clinician acceptance of telerehabilitation is key to service implementation,^40,41^ and highlights the core role of the ‘individual’ in implementation success. Even in the context of increased remote service capabilities in response to COVID-19,^42^ clinician reluctance may relate to feelings of limited preparation and training,^36,43^ or as identified by participants in this study be inherently linked to the training of healthcare professionals in a face-to-face environment with in-person patient interactions. In a survey of telehealth use among healthcare professionals conducted from November 2021–March 2022 of those who had experience using telehealth (*n* = 491), nearly half had less than 1-year experience.^44^ Whether healthcare professional acceptance of remote service delivery models will evolve with increased experience, and/or a future workforce who have received formal training in alternative service delivery, is not clear. The development of core capability frameworks, such as that proposed by Davies et al, to support skills training in the delivery of remote health may encourage greater confidence and acceptability.^43^

Key factors to support health professionals to deliver telerehabilitation for people with chronic lung disease were common across different healthcare contexts, suggesting broader telerehabilitation implementation strategies may be applicable across healthcare environments. Health professionals considered an online toolbox a valuable resource that could support individual clinician knowledge and skills and thus confidence and acceptance of remote PR delivery. However, organisational-level issues such as service funding and equipment and technology accessibility still exist. Despite increased remote healthcare service delivery in response to COVID-19, common barriers to the implementation of remote PR remain irrespective of health system or geographical location.

## Supplemental Material


Supplemental Material - Supporting delivery of remote pulmonary rehabilitation across different healthcare contexts: A multi-national study
Supplemental Material for Supporting delivery of remote pulmonary rehabilitation across different healthcare contexts: A multi-national study by Narelle S Cox, Sarah Rawlings, Natasha A Lannin, Sarah Candy, Surya P Bhatt, Abraham Samuel Babu and Anne E Holland in Journal of Chronic Respiratory Disease.
