# Supplementary material for: Supporting delivery of remote pulmonary rehabilitation across different healthcare contexts: A multi-national study
Source: Chron Respir Dis. 2024 Oct 7;21:14799731241290518. doi: 10.1177/14799731241290518 (PMC11459601; doi:10.1177/14799731241290518)
Supplement: Supplemental Material - Supporting delivery of remote pulmonary rehabilitation across different healthcare contexts: A multi-national study [file sj-pdf-1-crd-10.1177_14799731241290518.pdf]

Supporting delivery of remote pulmonary rehabilitation across different healthcare contexts: A multi-national study.

NS Cox, S Rawlings, NA Lannin, S Candy, SP Bhatt, AS Babu, AE Holland

Supplementary material

Results: TDF

'Environment, context and resources' was the most frequently coded TDF domain. This domain represents the circumstances of a person's situation or environment that promote (or impede) the development of skills or behaviours,[1, 2] and can relate to the patient, healthcare professional or the physical environment of either. Workforce capacity and the workplace and patient environment were key barriers to the delivery of remote rehabilitation. Insufficient staffing limited participants ability to provide remote rehabilitation service options alongside the traditional model of care. Likewise, a lack of appropriate infrastructure including technology eg. multiple screens of sufficient resolution and exercise equipment adaptable to the patients home environment contributed to difficulty transitioning to effective remote rehabilitation delivery. Limited access to technology was perceived by the health professionals as a significant barrier for patients across all healthcare contexts including no or unstable internet access and not having access to a device or a device with appropriate screen size for audio-visual communication whilst exercising. A safe location to exercise, pre-prepared exercise resources, access to low-technology exercise equipment e.g exercise bands, and ideally monitoring equipment that was readily available and easy to use for patients were seen as assisting in the implementation of remote rehabilitation.

The 'Knowledge' domain of the TDF pertains to the awareness of the existence of something [1, 2] and could relate to procedural, theoretical or physical knowledge. Poor digital literacy for both patients and health professionals were seen as impediments to remote rehabilitation delivery.

'Skills' refers to ability or proficiency attained through practice. In the context of remote rehabilitation delivery technology skills, together with communication and inter-personal skills, were reported as important features of healthcare professionals to deliver such programs.

Table S1 Focus group discussion guide

1. Can you describe for me what the pulmonary rehabilitation program looks like in your service currently?

- *Tell me about your role in the pulmonary rehabilitation team?*
- *Who are the people/skills that make up your pulmonary rehabilitation team/service?*
- *How often do patients attend/how long do patients attend a program for?*
- *How do you evaluate patient outcomes of pulmonary rehabilitation?*
- *How do you evaluate service outcomes of pulmonary rehabilitation?*

2. Can you describe for me your feelings about the concept of home-based pulmonary rehabilitation?

- *What are the things you think might be good about home-based pulmonary rehabilitation?*
- *What are the things you think might be difficult/bad/challenging about home-based pulmonary rehabilitation?*
- *What would be your expectations for patients if they undertook home-based pulmonary rehabilitation?*
- *What would be your expectations for your staff/yourself (professionally) if your site delivered a home-based pulmonary rehabilitation?*

3. Describe for me any previous experience you have of delivering pulmonary rehabilitation outside of your usual (ie. centre-based) model?

- *What did the 'other' pulmonary rehabilitation program you delivered look like?*
- *What resources were available to you to help deliver such a program?*
- *What, if any, features of the 'other' pulmonary rehabilitation program you were involved with might you change to make it easier in future?*
- *What, if any, features of the 'other' pulmonary rehabilitation program you were involved with would you keep?*

4. If you were to be involved in delivering a home-based pulmonary rehabilitation program at your site, what do you think would make you feel confident to deliver such a program?

- *What resources (human/physical/other) do you think would make it possible/easier to deliver a home-based pulmonary rehabilitation program?*
- *What resources/guidelines/recommendations/literature are you aware of that would support you to deliver a home-based pulmonary rehabilitation program?*
- *What challenges would you anticipate in trying to deliver a home-based pulmonary rehabilitation program?*

- *What do you think would make you feel competent to deliver a home-based pulmonary rehabilitation program?*
- *What might delivering a home-based pulmonary rehabilitation program mean for you in a professional capacity?*

5. If your site delivered/offered a home-based pulmonary rehabilitation program, what do you think might be the impact of doing this?

- *How do you think it might affect the patient/clients and their caregivers?*
- *How do you think it might affect your clinical role?*
- *How do you think it might affect other staff?*
- *How do you think it might affect your usual pulmonary rehabilitation service?*

6. Are there any other comments you would like to make?

Table S2: Interview guide

**Descriptive questions**	**Possible probing questions** (*These are a guide only. Depending on what the participant tells you, you do not have to ask all these questions or use the words exactly as written.)*
1. Can you briefly tell me about any remote models of pulmonary rehabilitation currently delivered at your site?	• *Can you tell me about any change to the model of pulmonary rehabilitation delivery at your site since we last spoke?*
2. Are there particular barriers or challenges that you face in being able to deliver pulmonary rehabilitation remotely at your site at the moment?	• *Have these barriers/challenges changed over time?*<br>• *Are there any resources/support you have accessed/found useful for addressing these barriers? That have made it easier to deliver remote pulmonary rehabilitation?*<br>• Resources in other languages: are you willing/able to share on the website
3. Can you describe your experience of using/viewing the prTelerehab website?	• What aspects of the prTelerehab website did you find good or useful?<br>• What aspects of the prTelerehab website did you find not useful or unhelpful?<br>• What things might you like to see more information about on the website?<br>• Resources??<br>• How did you find the functionality of the website?
4. When delivering a remote pulmonary rehabilitation program at your site, what makes you feel confident to deliver such a program?	• *What do you think would make [makes] you feel confident/ competent to deliver such a program?*
5. In your opinion, what could be [is] the impact of being able to deliver a remote home-based pulmonary rehabilitation program at your site?	• *How might [does] such a program affect the patient/clients and their caregivers?*<br>• *How might [does] such a program affect other staff?*

	• *How do you think such a program might [does] affect your usual pulmonary rehabilitation service?*
6. If a colleague asked you about delivering pulmonary rehabilitation remotely, what advice or recommendations would you offer them?	
7. Is there anything else you would like to say, that we have not talked about in this interview?	

References:

1. Cane J, O'Connor D and Michie S. Validation of the theoretical domains framework for use in behaviour change and implementation research. *Implement Sci* 2012; 7: 37. 2012/04/26. DOI: 10.1186/1748-5908-7-37.
2. Huijg JM, Gebhardt WA, Crone MR, et al. Discriminant content validity of a theoretical domains framework questionnaire for use in implementation research. *Implement Sci* 2014; 9: 11. 2014/01/16. DOI: 10.1186/1748-5908-9-11.
